# Diagnostic, Predictive, Prognostic, and Therapeutic Molecular Biomarkers in Third Millennium: A Breakthrough in Gastric Cancer

**DOI:** 10.1155/2017/7869802

**Published:** 2017-09-28

**Authors:** Nicola Carlomagno, Paola Incollingo, Vincenzo Tammaro, Gaia Peluso, Niccolò Rupealta, Gaetano Chiacchio, Maria Laura Sandoval Sotelo, Gianluca Minieri, Antonio Pisani, Eleonora Riccio, Massimo Sabbatini, Umberto Marcello Bracale, Armando Calogero, Concetta Anna Dodaro, Michele Santangelo

**Affiliations:** ^1^Department of Advanced Biomedical Science, University of Naples Federico II, Via S. Pansini 5, 80131 Naples, Italy; ^2^Department of Public Health, University of Naples Federico II, Via S. Pansini 5, 80131 Naples, Italy

## Abstract

**Introduction:**

Gastric cancer is the fifth most common cancer and the third cause of cancer death. The clinical outcomes of the patients are still not encouraging with a low rate of 5 years' survival. Often the disease is diagnosed at advanced stages and this obviously negatively affects patients outcomes. A deep understanding of molecular basis of gastric cancer can lead to the identification of diagnostic, predictive, prognostic, and therapeutic biomarkers.

**Main Body:**

This paper aims to give a global view on the molecular classification and mechanisms involved in the development of the tumour and on the biomarkers for gastric cancer. We discuss the role of E-cadherin, HER2, fibroblast growth factor receptor (FGFR), MET, human epidermal growth factor receptor (EGFR), hepatocyte growth factor receptor (HGFR), mammalian target of rapamycin (mTOR), microsatellite instability (MSI), PD-L1, and TP53. We have also considered in this manuscript new emerging biomarkers as matrix metalloproteases (MMPs), microRNAs, and long noncoding RNAs (lncRNAs).

**Conclusions:**

Identifying and validating diagnostic, prognostic, predictive, and therapeutic biomarkers will have a huge impact on patients outcomes as they will allow early detection of tumours and also guide the choice of a targeted therapy based on specific molecular features of the cancer.

## 1. Introduction

Gastric cancer is the fifth most common cancer after cancers of the lung, breast, colorectum, and prostate and it is the third cause of cancer death worldwide [1, 2]. The clinical outcomes of patient affected by gastric cancer are still not encouraging; indeed the 5 years' survival is less than 30% [3–5]. The incidence of gastric cancer is wildly different among the countries. Even though Japan has a higher incidence it also has a higher survival rate (52%) compared to other countries [4, 6, 7].

In 1965, Laurén classification of gastric cancer was introduced; it divides cancer into two types: intestinal and diffuse types which seem to have a different pathogenesis. The intestinal type is characterized by a cohesive and expansive growth pattern, it consists of neoplastic intestinal glands similar to the intestinal adenocarcinoma. The age of incidence of the intestinal type is higher than the diffuse type; it occurs more often in males and is more often located in the antrum; it predominates in high risk areas and is preceded by precancerous lesions. It is associated with* H. pylori *infection that leads to atrophic gastritis and intestinal metaplasia (precursor of intestinal type gastric cancer) [8–11]. The diffuse type is characterized by an infiltrative and noncohesive growth pattern with single neoplastic cell or small group of cells widely infiltrating the gastric wall. It occurs in younger patients, with no significant difference between men and women, and it is more often located in the gastric body [11, 12].

The TNM system is used for the staging of gastric cancer; however patients that belong to the same TNM stage often show very different clinical outcomes; this clearly manifests that there must be molecular factors responsible for those clinical differences.

A deep understanding of the molecular factors involved in the development of gastric cancer is needed in order to identify new biomarkers to diagnose GC in early stages and develop more effective therapeutic strategies.

**Figure 1:**
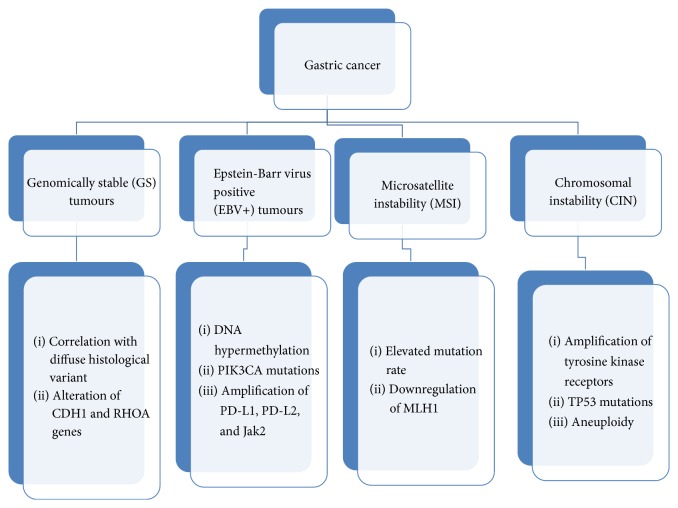
Classification of gastric cancers on the base of their molecular features (2014).

In 2014 gastric cancer has been classified into four subtypes on the base of their molecular features: genomically stable (GS) tumours; tumours characterized by chromosomal instability (CIN); tumours positive for Epstein-Barr virus (EBV-positive); tumours characterized by microsatellite instability (MSI-positive). The GS tumours, nearly diploid tumours, are correlated to diffuse histological variant and alterations of genes CDH1 and RHOA. The CIN tumours are characterized by focal amplification of tyrosine kinase receptors, TP53 mutations, and aneuploidy. The Epstein-Barr positive tumours are associated with high levels of DNA hypermethylation,* PIK3CA* mutations, and amplification of* CD274* (also known as* PD-L1*) and* PDCD1LG2* (also known as* PD-L2*) and JAK2. The MSI tumours display elevated mutation rate and downregulation of the MLH1 gene that codifies MLH1 protein involved in the mismatch repair (MMR) [13] (Figure 1).

It is essential to discover new biomarkers of gastric cancer that could lead to an early detection of the tumour or give predictive information about the response to a therapy and finally improve the therapeutic outcomes [14].

A valid biomarker for malignant tumour needs to have specific characteristics: it has to be detectable in high level in patient affected by cancer and undetectable or present in low level in people not affected; it has to be easily quantifiable in clinical sample; it has to show functions related to the progression of the disease and it has to provide prognostic or diagnostic information about the cancer [15, 16].

Biomarkers can be classified into four types: diagnostic, prognostic, predictive, and therapeutic. A diagnostic biomarker allows the early detection of the cancer in a noninvasive way and thus the secondary prevention of the cancer. A predictive biomarker allows predicting the response of the patient to a targeted therapy and so defining subpopulations of patients that are likely going to benefit from a specific therapy. A prognostic biomarker is a clinical or biological characteristic that provides information on the likely course of the disease; it gives information about the outcome of the patient [14, 17, 18]. A therapeutic biomarker is generally a protein that could be used as target for a therapy [18].

This paper aims to give a global view on the biomarkers for gastric cancer; we discuss the role of E-cadherin, HER2, fibroblast growth factor receptor (FGFR), MET, human epidermal growth factor receptor (EGFR), hepatocyte growth factor receptor (HGFR), mammalian target of rapamycin (mTOR), microsatellite instability (MSI), PD-L1, and TP53. We have also considered in this manuscript new emerging biomarkers as matrix metalloproteases (MMPs), microRNAs, and long noncoding RNAs (lncRNAs).

## 2. E-Cadherin

E-Cadherin is a transmembrane glycoprotein that is involved in the cellular calcium-mediated adhesion. It is codified by CDH1 located on the chromosome 16 (q22.1) [19, 20]. E-Cadherin plays a very important role in the adhesion and differentiation of the epithelial gastric cells and in the prevention of cancer onset [21]. CDH1 is one of the most important suppressor genes in gastric cancer; its inactivation increases tumour cells proliferation, invasion, and metastasis [18, 19, 22–25]. Several mechanisms can lead to loss of function of E-cadherin: mutations of the gene CDH1, loss of heterozygosis (LOH), silencing through suppressors that bind the promoter of CDH1 or through hypermethylation, and microRNAs that control the E-cadherin expression [20].

Analysing family affected, many germline mutations have been identified in hereditary diffuse gastric cancer [26]. Those mutations are spread in the 16 exons of the CDH1 gene; approximately 25% of them are missense mutations and 75% are truncating mutations [27, 28]. Only in a little percentage of family affected by gastric cancer (4%) have large deletions of CDH1 gene been identified [29, 30]. In the 70% of HDGC families germline there is a monoallelic mutation of the gene CDH1 that leads to a Loss of Heterozygosity (LOH) of the normal gene [31]. The cancer develops only when the second hit occurs, according to Knudson's model of the inactivation of tumour suppressor gene [31–34]. The second hit it is more often a hypermethylation of the gene's promoter and less often a second mutation or deletion occurring on the normal allele [21, 35–37]. The gastric cancer manifests when the complete inactivation of the CDH1 gene occurs, leading to a lack of the E-cadherin expression [27, 38]. Nowadays E-cadherin mutation cannot be considered a therapeutic biomarker as it would imply repairing E-cadherin expression through gene therapy [19]. Different E-cadherin alterations lead to various clinical manifestations and histotypes of gastric cancer so the presence of E-cadherin alteration is a weak prognostic biomarker [24]. A study showed a strong association between abnormal E-cadherin expression and tumour grade and metastases to regional lymph nodes [19]. Also another study showed the association between methylation of E-cadherin and dimension, stage of the cancer, and lymph nodes involvement [39]. Contrariwise another study did not find any association between E-cadherin mutation and gastric cancer stage and grade [40]. In most of the studies there is a correlation between E-cadherin abnormalities and worse clinical course, worse prognosis of the patient, and lower survival rate than patients negative for CDH1 mutations [25, 41].

E-Cadherin can also be considered as a predictive biomarker of the sensitivity to a specific therapy as its disablement reduces the response to both conventional and targeted therapy [24, 42]. Identifying CDH1 mutations at the moment of the diagnosis can predict if that cancer is going to be responsive to a therapy and so it could help in choosing the more suitable therapy for a specific patient [25] (Table 1).

It is important to highlight that a high percentage of families with HDGC have not got a mutation of E-cadherin gene; this obviously implies that there must be other molecular alterations that lead to the predisposition to gastric cancer and that still have not been identified [29, 43].

## 3. Microsatellite Instability

Microsatellites are short DNA repetitive sequences, in a nonrandom distribution along the human genome, that during DNA replication can lose out base-pairing mistakes [44, 45]. Those mistakes are normally repaired by the mismatch repair (MMR) proteins MLH1, MSH2, PMS2, and MSH6. Defects in the mismatch repair lead to a gathering of mutations that reflects the MSI and favour the onset of different types of cancer including the gastric one [46]. Several studies have reported an association between defects of mismatch repair and gastric cancer [47, 48]. MSI is observed in a percentage between 15 and 30 of all the gastric cancers and is more often due to hypermethylation of MLH1 promoter and the consequent lack of MLH1 expression [49–52]. MSI-positive gastric cancers show specific features: they usually have a later onset in life and are often located in distal part of the stomach and they usually have an intestinal histotype [45, 53–56]. The MSI in patients affected by gastric cancer seems to be a positive prognostic factor [57]. MSI-positive tumours show a better prognosis compared to MSI-negative as they have a lower local invasion capacity and have a lower prevalence of lymph nodes involvement; they also have a higher survival rate compared to MSI-negative gastric cancer at the same stage [50, 55–60] (Table 1).

## 4. PD-L1

PD-L1 and PD-L2 are ligands of Programmed Death-1 (PD-1) that is an important checkpoint receptor involved in the regulation of immunity and tolerance mechanism of T-cell. PD-L1 binding PD-1 is responsible for inducing and keeping the tolerance of peripheral T-cells [57]. PD-L1 is overexpressed in about the 40% of gastric cancer belonging to the EBV-positive type [13, 61]. Neoplastic cells use the PD-1/PD-L1 pathway to escape the immune surveillance of T-cells and the immune system reply to the cancer [62, 63].

A monoclonal antibody anti-PD-1, Pembrolizumab, has manifested efficacy in patients affected by advanced gastric cancer, showing a six-month OS of 69% [64]. The overexpression of PD-L1 can then be considered as a predictive biomarker of the response to a targeted therapy (Table 1). Targeting the PD1/PD-L1 pathway represents a promising strategy for the treatment of GC [57, 65].

## 5. TP53

p53 is a nuclear protein that works as a transcriptional factor whose duty is to keep the genomic stability. When a damage of the DNA occurs, p53 binds the DNA and activates the transcription of genes responsible for stopping the cellular cycle and causing apoptosis of the cell. p53 is encoded by the gene TP53 located on the chromosome 17p13.1 [11, 66, 67]. The mechanisms leading to the damage of TP53 function are usually LOH and mutations and less often methylation [68]. TP53 mutation is frequently mutated in gastric cancer and it is reported in association with CIN subtype [13, 68]. Heterogeneity of TP53 mutations in the same tumour is also reported as a result of multiple mutations of the gene [68]. Studies reported a higher prevalence of TP53 mutations in the intestinal type than in the diffuse type; another study instead reported a similar prevalence of TP53 mutations in the two types. Early and advanced intestinal type as well as advanced diffuse type show a high similar prevalence of TP53 mutations that are instead infrequent in early diffuse type of gastric cancer [68–72]. A correlation between p53 overexpression and size of the gastric cancer has been reported [73]. The association between p53 overexpression with lymph nodes metastasis and shorter survival is still controversial because it has been reported in some studies but not in others; therefore, at this moment in time, p53 cannot be considered a trustworthy prognostic biomarker [57, 68] (Table 1).

## 6. HER2

HER2 is one of the four tyrosine receptor kinases (RTKs) belonging to the family of EGFR (EGFR or HER1, HER2, HER3, and HER4); it is codified by the protooncogene ERBB2 located on chromosome 17q21 and plays an important role in cell survival and proliferation [14, 74].

For signal transmission HER2 needs to heterodimerize with another member of the HER family, mainly with EGFR [75]. The amplification of ERBB2 gene produces an overexpression of HER2 protein that leads to cancer cells survival, growth, and proliferation through the PI3K-AKT and the MAPK pathways [76, 77]. Overexpression of HER2 receptor as a prognostic and predictive biomarker, identified before in breast cancer, is becoming noticeable even in gastric cancer [57]. HER 2 overexpression has a variable incidence ranging from 9% to 38% in most of the studies, depending on the location of the cancer and on its histology [76, 78–82]. The HER2 overexpression is more frequently observed in gastroesophageal junction tumours than in those with distal gastric location and it is more often associated with the intestinal type adenocarcinomas [83–90]. Cancers positive for HER2 overexpression are usually differentiated tumours [43, 80, 91]. ERBB2 gene mutation that leads to HER2 overexpression occurs in the early stage of carcinogenesis [92].

The role as a prognostic biomarker of HER2 is still doubtful; indeed some studies show an association of HER2 with a worse prognosis and a more aggressive disease; others contrariwise do not find a significant difference in prognosis between HER2 positive and HER2 negative cancers [76, 80–82, 91, 93–103].

Still controversial is also the correlation between HER2 overexpression and clinical features of the tumour. Some studies indeed suggest an association of ERBB2 amplification with tumour size, lymph node metastasis, local invasion, and cancer stage; other studies instead do not find any link between them [85, 87–90, 95, 100, 102].

HER2 overexpression has become a very important predictive biomarker that allows clinicians to identify patients that are going to have a survival benefit from a biological therapy with the monoclonal antibody (trastuzumab) [104–106].

The ToGa clinical phase 3 randomized controlled trial, conducted on patient affected by advanced gastric or gastroesophageal junction cancer, HER2-positive with an immunohistochemical 3+ score, compared the effectiveness of the association of trastuzumab and chemotherapy (cisplatin and a fluoropyrimidine) with the chemotherapy alone. The results of this study pointed out that patients treated with the association of trastuzumab and chemotherapy had a longer OS (13.8 months versus 11.1) and even their progression free survival (PFS) was heightened compared to that of the patient treated only with chemotherapy [104].

At the moment, trastuzumab is the only targeted therapy permitted for advanced gastric cancer [107]. Other ways of blocking the HER2 receptor are now being researched.

Lapatinib is a tyrosine kinase inhibitor that blocks both HER2 and EGFR. A randomized phase III TyTAN trial compared the efficacy of the association lapatinib and paclitaxel with paclitaxel alone, in patients affected by HER2-positive advanced gastric cancer. The OS was of 11.0 months in patient treated with the association of lapatinib and paclitaxel and 8.9 months in the ones treated with paclitaxel alone and also the response rate was increased with the associated therapy, yet there was no significant difference in PFS [108].

Other HER2 targeted drugs such as neratinib and pertuzumab, whose efficacy on HER2-positive breast cancer has already been proved, have not been assessed yet on advanced gastric cancer in randomized clinical trials [57].

Ado-trastuzumab emtansine (T-DM1) is a drug composed of the monoclonal antibody trastuzumab linked to a cytotoxic drug on microtubules DM1. This conjugate efficacy has been evaluated in the phase II/III Gatsby, whose results have not been released yet, but ImmunoGen has revealed that they are not encouraging [109].

HER2 can then be considered as an important predictive biomarker that can guide the choice of the best therapy for the single patient (Table 1).

## 7. EGFR

Even EGFR belongs to the family of tyrosine kinase receptors. It was found to be overexpressed in about the 27% of gastric cancer and the incidence of the amplification of the gene was from 3% to 8% depending on the detection method used [110–112].

EGFR overexpression has been related to cancer histology slightly differentiated, low survival, and high stage [111].

Unfortunately, the use of targeted therapy anti-EGFR (cetuximab or panitumumab) together with chemotherapy did not show any improvement in the outcomes of the patients affected by advanced gastric cancer [113, 114] (Table 1).

Even inhibitors of tyrosine kinase (TKIs) have been considered as therapy in patients affected by advanced gastric cancer resistant to chemotherapy [78].

Unluckily, none of the studies has demonstrated a significant improvement of results compared to conventional therapy. Considering premises already made, further investigations are needed to identify subgroups of patients that might benefit from anti- EGFR therapies.

## 8. FGFR

FGFR1, FGFR2, FGFR3, and FGFR4 are fibroblast growth factor receptors belonging to the RTK family [14]. In 2012, Deng et al. reported that FGFR2 copy number gain was detected in 9% of cancers [110]. Considering the high expression of this receptor in some tumours, phase II studies are evaluating the efficacy of dovitinib (TKI258), a small FGFR2 inhibitor, on patients with FGFR2 amplification positive gastric cancer [110].

## 9. mTOR

The activation of many RTKs induces the activation of phosphatidylinositol-3-kinase (PIK3)/mTOR pathway. Mutations of the gene PIK3CA that codifies the alpha p110 catalytic subunit of PIK3 lead to constitutive activation of the PIK3/mTOR pathway [14, 115]. PIK3CA mutation has been associated with a worse prognosis with reduced survival and increased lymph node metastasis [14, 116] (Table 1). The frequency of mutations varies from 5 to 67% in different studies [117–120]. PIK3CA mutations frequently occur in EBV-positive gastric cancer [119].

A mTOR inhibitor, Everolimus, has displayed potential benefit in advanced gastric cancer in phase II trials; however in phase III trials it did not lead to any significant rising of OS [121–123].

## 10. MET

MET is a RKT belonging to the family of hepatocyte growth factor receptor (HGFR), it binds HGF/SF (hepatocyte growth factor/scatter factor). Autophosphorylation of MET leads to the activation of a number of downstream pathways (PIK3, Akt, and RAS-MAPK) responsible for cancer cell survival, proliferation, invasion, and metastasization [124].

It is overexpressed in about 50% of advanced gastric cancer [65, 125].

MET gene overexpression is related to a bad prognosis; it is associated with a more aggressive disease, a shorter OS, and disease free survival compared to MET-negative gastric cancers [126–129].

It is also an important predictive biomarker. Rilotumumab is a monoclonal antibody able to prevent the binding of MET receptor and its ligand HGF; this targeted therapy in association with the chemotherapy improves the OS to 11.1 months in patient affected by a high level MET amplification cancer compared with 5.7 months of the patient that received the chemotherapy alone [130] (Table 1).

MET importance on carcinogenesis is becoming so evident that, nowadays, multiple studies are evaluating the efficacy of TKIs (like crizotinib and foretinib) on cancers with MET overexpression [125, 131].

## 11. Promising Future Markers 

### 11.1. Matrix Metalloproteinase

The matrix metalloproteinases (MMPs) are a family of zinc-dependent endoproteinase whose function is to degrade the elements of the extracellular matrix [132]. Their work is regulated by the inhibitors of metalloproteinase (TIMPs) [133]. MMPs are involved in many physiological and also pathological processes [134]. MMPs have been found upregulated in gastric cancer and they have also been associated with specific pathological features of the cancer. Studies conducted on this subject prompt that MMPs and TIMP could be used as markers of peritoneal dissemination, depth of invasion, and metastasis [132].

Unfortunately, MMPs inhibitors have not demonstrated significant clinical benefit as therapy. In a clinical trial, conducted on patients affected by chemotherapy refractory advanced gastric cancer and gastroesophageal cancer, the MMP marimastat only determined a little difference in survival. The treatment was burdened by low tolerability and musculoskeletal pain [135]. Further studies about this subject are needed in order to identify the possible application of MMP in therapy of the advanced gastric cancer.

### 11.2. MicroRNA

MicroRNAs are 18 to 24 nucleotides noncoding RNA fragments whose function is to bind the 3′UTR region of their target gene and regulate its expression by impairing the translation [136–138]. MicroRNAs are involved in the regulation of several process of the cell: proliferation, differentiation, migration, and invasion [136]. Many genes can be regulated just by a microRNA [139]. MicroRNAs seem to play a very important role in the carcinogenesis of gastric cancer; they can increase the expression of oncogenes or reduce the expression of tumour suppressor genes [139, 140].

Several microRNAs have been identified and recognized to be implicated in gastric cancer [141, 142]. It is difficult to pick a miRNA as a cancer biomarker. Currently, there are no studies proving the effectiveness of miRNAs as predictive, prognostic or therapeutic biomarkers [57].

### 11.3. Long Noncoding RNAs

Long noncoding RNAs (lncRNAs) are sequences of nucleotides longer than 200 [143, 144]. Currently lncRNAs are catching researchers' attention because of an increasing amount of evidence suggesting that they play an important role in carcinogenesis and metastasis [139]. Nowadays about 135 lncRNAs have been recognized as altered in gastric cancer, so their potential role as diagnostic and prognostic markers has been speculated [143–145]. However, further studies about lncRNAs are needed in order to identify their possible clinical utilization.

## 12. Conclusions

Even if the incidence of gastric cancer reduced, it still remains the fifth most common cancer and it is characterized by negative prognosis and bad outcomes in response to chemotherapy. A deep understanding of molecular mechanisms of gastric carcinogenesis is essential to develop new therapeutic strategies and diagnostic, prognostic, and predictive biomarkers. The partition of gastric cancer into four molecular types (EBV-positive, MSI-positive, genomically stable, and chromosomal instability) allows dividing the patients on the basis of the molecular features of their cancer and identifying the best therapeutic approach [13]. A huge amount of studies has been conducted on molecular biomarkers; however the only predictive biomarker currently used is HER2 that allows identifying the patient that will benefit from a targeted therapy with trastuzumab. The majority of the patients still cannot be treated with a targeted therapy and nowadays still there are no diagnostic markers that can be used for secondary prevention. Most of the biomarkers till now identified still need to be validated before they can actually be employed in clinical practice [14]. Further studies that will identify and validate diagnostic, prognostic, predictive, and therapeutic biomarkers will have a huge impact on the outcomes of the patients, as they will allow the early detection of the tumour and also guide the choice of a targeted therapy based on the specific molecular features of the cancer [146–149].

## Figures and Tables

**Table 1:** 

Biomarkers characteristics	Prognostic value	Predictive value
*E-Cadherin* Transmembrane glycoprotein involved in calcium mediated adhesion, codified by gene CDH1 (chromosome 16q22.1). Its alterations lead to increased cell proliferation, invasion, and metastasis.	It is associated with worse prognosis and lower survival rate	It is associated with reduced response to conventional and targeted therapy

*Microsatellite instability* Microsatellites are short repetitive sequences that can lose out base paring during replication. Defects in mismatch repair lead to MSI. Tumours with MSI usually have (i) later onset (ii) distal location (iii) intestinal histotype.	MSI-positive cancers are associated with (i) a better prognosis than MSI-negative (ii) higher survival rate (iii) lower local invasion capacity (iv) lower prevalence of lymph nodes involvement	—

*PD-L1* It is the ligand of Programmed Death-1; it is responsible for inducing and keeping tolerance of peripheral T-cells. PD-1/PD-L1 pathway is used by neoplastic cell to escape immune surveillance.	—	PD-L1 overexpression is a predictive biomarker of response to Pembrolizumab

*TP53* P53 is a nuclear protein that works as a transcriptional factor that activates apoptosis in case of DNA damage. It is codified by TP53 (chromosome 17p13). P53 alterations are often associated with CIN subtype.	There is a correlation between p53 overexpression and tumour size. The association with lymph nodes metastasis and shorter survival is still controversial	—

*HER2* It is a tyrosine receptor kinase (RTK) belonging to EGFR family, codified by ERBB2 (chromosome 17q21). It is involved in cell survival and proliferation. HER2+ tumours are often located at the gastroesophageal junction and often associated with intestinal histotype.	Still controversial: some studies report a more aggressive disease with worse prognosis but other studies do not confirm it	It is a predictive biomarker of the response to trastuzumab and lapatinib

*EGFR* It belongs to the family of tyrosine kinase receptors.	It is associated with slightly differentiated, to high stage tumours and to a low survival	The use of anti-EGFR (cetuximab and panitumumab) associated with chemotherapy did not show any improvement in the clinical outcomes

*FGFR1-4* The fibroblast growth factor receptors belong to RTK family.	Under evaluation	Under evaluation

*mTOR* The activation of many RTK induces the activation of PIK3/mTOR pathway. PIK3CA mutations frequently occur in EBV positive cancers.	PIK3CA mutation has been associated with (i) worse prognosis (ii) reduced survival rate (iii) increased lymph nodes metastasis	Constitutive activation of PIK3/mTOR pathway is predictive of the response to Everolimus

*MET* It is a RKT belonging to the family of Hepatocyte Growth Factor Receptors (HGFR); it binds HGF/SF. Autophosphorylation of MET leads to the activation of downstream pathways responsible for cancer cells survival, proliferation, invasion, and metastasization.	It is associated with (i) more aggressive disease (ii) shorter survival	It is an important predictive biomarker of the response to rilotumumab
